# Supplementary material for: Engel’s law in the commodity composition of exports
Source: Sci Rep. 2019 Nov 1;9:15871. doi: 10.1038/s41598-019-52281-8 (PMC6825129; doi:10.1038/s41598-019-52281-8)
Supplement: Supplementary file 1 — Supplementary information [file 41598_2019_52281_MOESM1_ESM.pdf]

Supplementary Information:
Engel's law in the commodity composition of exports

Sung-Gook Choi[1] and Deok-Sun Lee[1]

[1] *Department of Physics, Inha University, Incheon 22212, Korea*

I. GDP-CORRELATED COMMODITIES AT TWO-DIGIT LEVEL

With 93 commodity categories obtained by keeping the first two digits of the SITC, we have investigated the relation between the shares of such refined commodity categories in the export values and the normalized GDP of countries and present the results in Fig. S1.

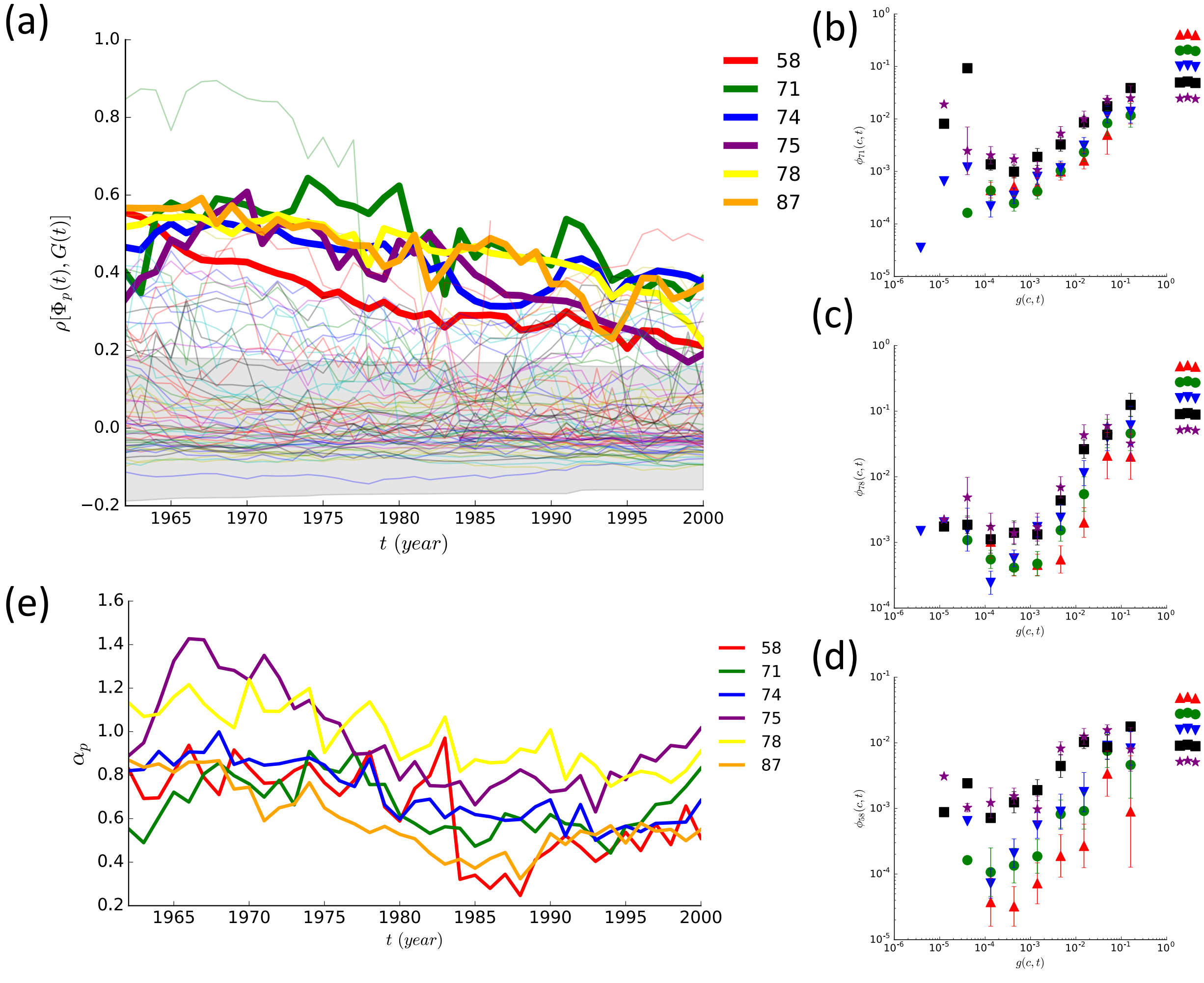

FIG. S1. Correlation between the GDP profile and the export share profile with 93 commodity categories based on the first two digits used. (a) The Pearson correlation coefficient in Eq. (4) between the local export share profile of a commodity and the GDP profile as a function of time t. The range of ρ with $P > 0.05$ is shaded. Thick lines are used for the six categories showing correlations with $P < 0.05$. (b) Plot of the share $\phi_{71}(c,t)$ of the *Power-generating machinery and equipment* category versus the normalized GDP $g(c,t)$ for selected years. (c) Plot of the share $\phi_{78}(c,t)$ of the *Road vehicles* category versus the normalized GDP. (d) Plot of the share $\phi_{58}(c,t)$ of the *Artificial resins and plastic materials, and cellulose esters etc* category versus the normalized GDP. (e) GDP-elasticity $\alpha_p(t)$ of the six GDP-correlated categories.

year (t)		1962					2000						
Cluster(C)		*0*	*20*	*2*	*3*	*76*	*026*	*20*	*3*	*36*	*76*	*87*	*8*
No. countries		31	28	17	9	25	18	10	17	6	31	15	7
GDP($g(C,t)$)		0.11	0.18	0.013	0.026	0.67	0.036	0.0029	0.034	0.041	0.61	0.23	0.0054
Export commodity portfolio	$\phi_0(C,t)$	0.618	0.320	0.076	0.018	0.108	0.282	0.132	0.017	0.069	0.049	0.056	0.122
	$\phi_1(C,t)$	0.018	0.025	0.001	0.000	0.017	0.016	0.019	0.001	0.002	0.009	0.005	0.022
	$\phi_2(C,t)$	0.164	0.414	0.698	0.032	0.105	0.186	0.486	0.011	0.060	0.030	0.036	0.015
	$\phi_3(C,t)$	0.032	0.097	0.034	0.932	0.042	0.149	0.048	0.873	0.458	0.039	0.053	0.002
	$\phi_4(C,t)$	0.023	0.020	0.017	0.000	0.008	0.011	0.009	0.000	0.001	0.003	0.004	0.001
	$\phi_5(C,t)$	0.024	0.016	0.006	0.001	0.077	0.048	0.023	0.033	0.062	0.101	0.042	0.005
	$\phi_6(C,t)$	0.052	0.080	0.142	0.010	0.252	0.164	0.079	0.033	0.219	0.138	0.189	0.084
	$\phi_7(C,t)$	0.044	0.011	0.017	0.003	0.305	0.073	0.067	0.020	0.037	0.496	0.260	0.051
	$\phi_8(C,t)$	0.020	0.008	0.006	0.001	0.071	0.032	0.127	0.008	0.025	0.107	0.340	0.684
	$\phi_9(C,t)$	0.004	0.010	0.004	0.002	0.015	0.038	0.011	0.003	0.068	0.029	0.015	0.014

TABLE S1. The number of countries, the normalized GDP, and the export commodity portfolio of five clusters in 1962 and seven clusters in 2000, as identified in Fig. 4 (a), are shown.

$C^{(1962)}$ \ $C^{(2000)}$	*026*	*20*	*3*	*36*	*76*	*87*	*8*
0	0.377	0.594	1.046	0.727	0.754	0.709	0.844
20	0.263	0.246	0.927	0.588	0.694	0.632	0.816
2	0.570	0.265	1.092	0.775	0.834	0.783	0.967
3	0.861	1.013	0.076	0.531	1.039	0.995	1.162
76	0.336	0.488	0.918	0.505	0.247	0.296	0.695

TABLE S2. The Euclidean distance between the average portfolios of the clusters of 1962 and 2000.

II. PROPERTIES OF THE COUNTRY CLUSTERS

Some properties of each cluster, including the average share of each category are given in Table S1. The Euclidean distance between the average portfolios of each pair of clusters of 1962 and 2000 is shown in Table S2.

Time-evolution of the GDP of countries classified by their transition behaviors

The time-evolution of the normalized GDP of all countries classified according to their transition between the clusters of 1962 and 2000 is shown in Fig. S2, which is an extended version of Fig. 5 and consists of five panels each for the countries starting at the same cluster in 1962 and arriving at different clusters in 2000.

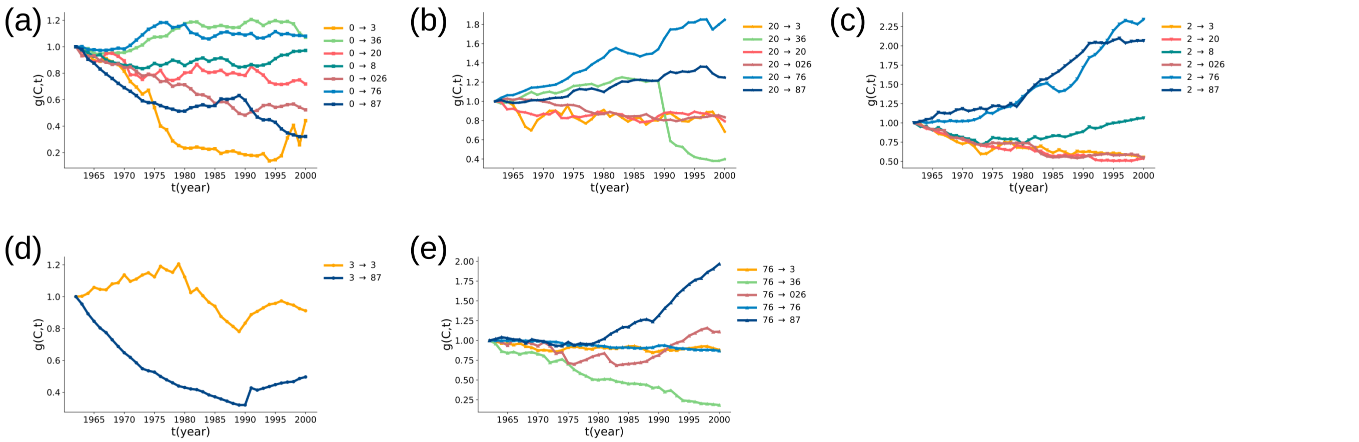

FIG. S2. The time evolution of the normalized GDP of countries classified according to their transition between clusters in 1962 and 2000. For the countries transiting from a cluster C in 1962 to a cluster C' in 2000, we plot their normalized GDP divided by that in 1962, $g(C \to C', t)/g(C \to C', 1962)$ as a function of time. The shape of data points varies with the cluster of 1962 and the color varies with the cluster of 2000. (a) The time evolution of the rescaled GDP $g(\mathit{0} \to C', t)/g(\mathit{0} \to C', 1962)$ for the countries which are in the cluster $\mathit{0}$ in 1962 and transit to a cluster C' in 2000. (b) Plots of $g(\mathit{20} \to C', t)/g(\mathit{20} \to C', 1962)$ versus time t for different clusters C' in 2000. (c) $g(\mathit{2} \to C', t)/g(\mathit{2} \to C', 1962)$ versus time t. (d) $g(\mathit{3} \to C', t)/g(\mathit{3} \to C', 1962)$ versus time t. (e) $g(\mathit{76} \to C', t)/g(\mathit{76} \to C', 1962)$ versus time t.
